# Extended-Spectrum β-Lactamase–Producing Enterobacterales in Municipal Wastewater Collections, Switzerland, 2019–2023

**DOI:** 10.3201/eid3103.240099

**Published:** 2025-03

**Authors:** Lisandra Aguilar-Bultet, Elena Gómez-Sanz, Ana B. García-Martín, Monica Alt Hug, Reto Furger, Lucas Eichenberger, Claudia Bagutti, Sarah Tschudin-Sutter

**Affiliations:** University Hospital Basel, Basel, Switzerland (L. Aguilar-Bultet, E. Gómez-Sanz, Ana B. García-Martín, S. Tschudin-Sutter); State Laboratory Basel-City, Basel (M. Alt Hug, R. Furger, L. Eichenberger, C. Bagutti)

**Keywords:** bacteria, antimicrobial resistance, ESBL-producing Enterobacterales, Escherichia coli, Klebsiella pneumoniae, COVID-19 pandemic, spatiotemporal distribution, municipal wastewater, ESBL-PE abundance, extended-spectrum β-lactamase, Switzerland

## Abstract

We quantified presumptive extended-spectrum β-lactamase–producing *Escherichia coli* and *Klebsiella*, *Enterobacter*, *Serratia*, and *Citrobacter* group colonies from wastewater in Basel, Switzerland, across 3 years to represent before, during, and after the COVID-19 pandemic. Wastewater surveillance might be a noninvasive, sensitive, rapid, and cost-effective instrument for early detection and monitoring local epidemiology.

Extended-spectrum β-lactamase–producing Enterobacterales (ESBL-PE), particularly *Escherichia coli* and species of the *Klebsiella*, *Enterobacter*, *Serratia*, and *Citrobacter* (KESC) group, are bacteria that contribute to the global antimicrobial resistance burden (*1*–*3*). A comprehensive understanding of the sources of the bacteria and relevant expansion factors is imperative.

The COVID-19 pandemic caused unprecedented challenges at a global scale by overwhelming healthcare systems and causing disruption of infection control and preventive measures (*4*,*5*). Antibiotic drugs were overprescribed for patients with COVID-19 because of concerns about secondary infections and co-infections (*4*,*6*,*7*). Such overuse of antibiotic drugs might have caused the emergence of multidrug-resistant pathogens. However, measures such as general lockdowns, social distancing, vaccination, reduced antibiotic drug use in the outpatient sector, extensive implementation of hand hygiene and face masks, decreased elective hospital procedures, and limited global international travel and migration reduced local antimicrobial resistance severity during the pandemic (*8*). 

Returning travelers have been recorded as a source of ESBL-PE (*9*). Thus, the influence of COVID-19 on ESBL-PE remains unclear, and surveillance data from clinical samples might be biased because of reduced patient care during the pandemic. In contrast, wastewater surveillance is independent of diagnostic tests and is increasingly recognized as a tool to track circulating bacterial and viral pathogens and drug resistance determinants (*10*,*11*). Here, we leveraged and applied an established wastewater surveillance system (*12*) to assess changes in the number of presumptive ESBL-producing *E. coli* and KESC in municipal wastewater before (2019), during (2021), and after (2023) the COVID-19 pandemic in Basel, Switzerland.

## The Study

We collected 125 wastewater samples distributed across Basel, Switzerland, for 3 consecutive months (April–June) in 2021 (n = 62 [33.0% of samples]) and 2023 (n = 63 [33.5% of samples]) using a systematic approach (Appendix) (*12*). To represent the baseline, we retrospectively included the quantification results of presumptive ESBL-producing *E. coli* and KESC of wastewater samples collected from the same wastewater sampling sites during a 12-month period covering 2018–2019. Those samples were collected as part of an earlier study on ESBL-PE in municipal wastewater and used the same sampling approach and methods (*12*) (Appendix). For the primary analyses, we chose not to incorporate all results from that earlier study because of differences in the timing of sample collection (Appendix Table 1). We analyzed samples collected within the same months (April–June) throughout the study period because we previously found differences in ESBL-PE counts across sampling months when analyzing ESBL-PE counts across an entire year (*12*).

Municipal wastewater samples were taken at 21 different sewer sampling points representing the 10 postal codes of Basel, as previously described (*12*). We categorized sites as urban (81.0%, n = 17), representing a community without wastewater from healthcare settings; and mixed (19.1%, n = 4), both community and healthcare settings (Appendix Table 2). The samples were collected by the Civil Engineering Department of the Canton of Basel-Stadt following the recommendations of the World Health Organization and processed as previously detailed (*12*).

We assessed differences in the number of presumptive ESBL-producing *E. coli*, KESC, and *E. coli* plus KESC by using the Friedman test, which is a nonparametric alternative to the repeated-measures analysis of variance and Kruskal-Wallis test for comparisons stratified by month. We compared urban versus mixed sites per sampling year for each bacterial group by using the Mann-Whitney U test. To account for the repeated testing of the sampling sites, we calculated the mean of 3 CFU/mL counts measured over the 3-month sampling period per sampling site and year (Appendix Table 3). We used Stata version 16.1 (StataCorp LLC, https://www.stata.com) to perform analyses. Reported p values are 2-sided and considered significant at <0.05. 

Median total counts combined across the 3 years were as follows: 140 CFU/mL, presumptive ESBL *E. coli*; 70 CFU/mL, KESC group; and 270 CFU/mL, *E. coli* plus KESC. For all 3 comparisons, we observed significant differences (p<0.001) (Table 1; Figure, panels A–C). For analyses stratified by the 3 sampling months, median counts statistically differed for May and June (p<0.05) (Appendix Table 4). For the data stratified by mixed (n = 35 [18.6% of samples]) versus urban (n = 153 [81.4% of samples]) sites, sites that included hospital sewage had higher counts overall (Figure 1, panels D–F). We detected larger values of presumptive ESBL-producing KESC colonies in sampling sites including hospital sewage compared with sampling sites receiving only community wastewater (2023 median counts: 620 CFU/mL mixed, 40 CFU/mL urban; p = 0.025) (Table 2; Figure 1, panels D–F), whereas Mann-Whitney U tests showed no statistically significant differences for other comparisons. Analyses stratified per month supported higher counts for mixed compared with urban sites during and after the pandemic (Appendix Table 5, Figures 1–4). 

**Table 1:** Quantification over 3 years of presumptive extended-spectrum β-lactamase–producing Enterobacterales in municipal wastewater collections, Switzerland, 2019–2023*

Colony	2019, n = 63			2021, n = 62			2023, n = 63	
	Median CFU/mL (IQR)	Range, CFU/mL		Median CFU/mL (IQR)	Range, CFU/mL		Median CFU/mL (IQR)	Range, CFU/mL
ESBL *Escherichia coli*	60 (8−180)	0–15,480		193 (50−520)	0−18,100		195 (58−708)	0−125,300
ESBL KESC	10 (0−83)	0−9,700		178 (51−731)	0−11,400		110 (13−373)	0−5,550
ESBL *E. coli* + KESC	120 (28−283)	0−15,480		473 (191−1,403)	0−11,400		475 (80−1,585)	0−125,300

*Comparisons for 2019 versus 2021 versus 2023 by Friedman test. All colony p values were statistically significant at <0.001. ESBL, extended-spectrum β-lactamase; IQR, interquartile range; KESC, *Klebsiella*, *Enterobacter*, *Serratia*, and *Citrobacter* group.

**Figure:**
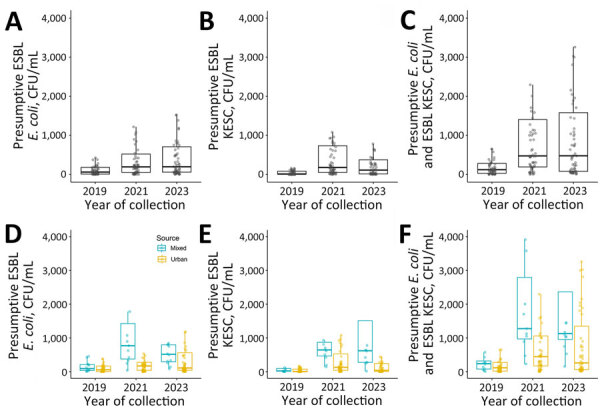
Temporal quantification of extended-spectrum β-lactamase–producing Enterobacterales in municipal wastewater collections, Switzerland, 2019–2023. A–C) Temporal distribution of presumptive ESBL-producing *Escherichia coli* (A), presumptive ESBL-producing KESC (B), and presumptive ESBL-producing *E. coli* plus KESC (C). Data from the 3-month sampling across the 21 sampling points distributed across Basel, Switzerland (representing 44% of the Basel population), are collapsed and represented by year: 2019, n = 63; 2021, n = 62; 2023, n = 63. Friedman test p values for all categories are <0.001. Quantification stratified by sample source. D–F) Temporal distribution by source, urban or mixed (community and hospital) effluents, of presumptive ESBL-producing *E. coli* (D) presumptive ESBL-producing KESC (E), and presumptive ESBL-producing *E. coli* plus KESC (F). Data from the 3-month sampling across the 17 urban (n = 51 per year) and 4 mixed (n = 12 per year, except for 1 data point missing in April 2021) sampling points are combined and represented per year. Box tops and bottoms indicate interquartile ranges; bold lines, medians; whiskers, 1.5 times the interquartile range. Jitter plots indicate individual data points. Outliers were removed for readability. Mann-Whitney sum U test p values stratified by mixed versus urban samples per chromogenic group (per year): *E. coli,* p = 0.654 (2019), 0.107 (2021), 0.371 (2023); KESC: 0.420 (2019), 0.179 (2021), 0.0251 (2023); *E. coli* plus KESC, p = 0.531 (2019), 0.283 (2021), 0.128 (2023). ESBL extended-spectrum β-lactamase; ESBL-PE, extended-spectrum β-lactamase–producing Enterobacterales; KESC, *Klebsiella, Enterobacter, Serratia,* and *Citrobacter.*

**Table 2:** Quantification across 3 years of presumptive extended-spectrum β-lactamase–producing Enterobacterales in municipal wastewater collections, Switzerland, 2019–2023, stratified by source*

Characteristics	ESBL *E. coli*			ESBL KESC			ESBL *E. coli* + KESC	
	Mixed	Urban		Mixed	Urban		Mixed	Urban
2019								
Sample size	n = 12	n = 51		n = 12	n = 51		n = 12	n = 51
Median CFU/mL (IQR)	90 (36−210)	55 (5−165)		23 (13−94)	5 (0−75)		238 (79−318)	115 (18−273)
Range, CFU/mL	5−455	0−15,480		0−840	0−9,700		5−885	0−15,480
p value	0.654			0.420			0.531	
2021								
Sample size	n = 11	n = 51		n = 11	n = 51		n = 11	n = 51
Median CFU/mL (IQR)	770 (375−1,428)	170 (40−278)		645 (465−870)	130 (40−528)		1,275 (970−2,793)	445 (170−1,058)
Range, CFU/mL	40−4,200	0−18,100		140−3,900	0−11,400		230−8100	0−18,300
p value	0.107			0.179			0.283	
2023								
Sample size	n = 12	n = 51		n = 12	n = 51		n = 12	n = 51
Median CFU/mL (IQR)	515 (296−798)	110 (43−563)		620 (275−1,511)	40 (10−243)		1,130 (956−2,365)	260 (63−1,348)
Range, CFU/mL	85−3,490	0−125,300		15−5,550	0−3,830		155−7,195	5−125,300
p value	0.371			**0.025**			0.128	

*Mixed versus urban by Mann-Whitney U test. Bold text indicates statistically significant value. ESBL, extended-spectrum β-lactamase; IQR, interquartile range; KESC, *Klebsiella*, *Enterobacter*, *Serratia*, and *Citrobacter* group.

One limitation of the study was the small number of wastewater samples, and we acknowledge that the prepandemic phase was represented by samples collected within 12 months spanning 2018 and 2019. To ensure comparability between the sample years, the main comparisons were made for April, May, and June because data were only available for those 3 months in 2019, 2021, and 2023 (Appendix Table 1). The samples collected in 2019 might not accurately reflect the baseline presence of ESBL-PE before the pandemic. Also, collection of specimens in 2020 and 2022 could have further substantiated a conclusion that the changes were caused by factors related to the pandemic. Another limitation of the study was that we could not collect samples in 2020 and 2022 because of a lack of resources. 

## Conclusions

Our wastewater surveillance showed increased presumptive ESBL-producing *E. coli* and KESC counts during and after the COVID-19 pandemic compared with the prepandemic period. Prevalence of ESBL-producing *Klebsiella pneumoniae* in humans has doubled in Switzerland since 2019, particularly in the northwest region where Basel is located (5% in 2019; 8% in 2021; 11% in 2023) (*13*). Highest presumptive ESBL-KESC counts in wastewater in 2021 might be related to the overwhelming global situation in the healthcare system, implying predominance of hospital-acquired infections, such as those caused by ESBL-producing *K. pneumoniae* (*14*,*15*). We observed an increase of presumptive ESBL-producing *E. coli* during 2021, without a subsequent decrease in 2023. Of note, the overall ESBL-producing *E. coli* resistance rates in humans in Switzerland have remained stable since 2015 (10%–11%) (*13*).

Our results suggest that the COVID-19 pandemic exacerbated the differences in ESBL-PE abundance between urban and mixed sites. That increase might be caused by the rise in ESBL-PE prevalence in local hospitals compared with before the pandemic, as is the case for ESBL *K. pneumoniae*, together with a slight increase detected in consumption of third- and fourth-generation cephalosporins in German-speaking Switzerland in the inpatient sector (defined daily dose per 100 bed-days: 2019, 6.5; 2020, 7.0; 2021, 6.8; 2022, 7.0) (*13*). Social distancing, travel restrictions, and decreases in third- and fourth-generation cephalosporin use (defined daily dose per 1,000 inhabitants per day: 2019, 0.05; 2021, 0.03) observed in the outpatient sector in the region during the years of the COVID-19 pandemic (*13*) might have promoted the disparity of presumptive ESBL-KESC abundance between urban and mixed wastewater samples in 2023.

In summary, we showed an increase in presumptive ESBL-producing *E. coli* and KESC in 2021 and 2023, particularly in samples containing hospital wastewater, suggesting a disproportionate increase of ESBL KESC within healthcare settings compared with the community and possibly explained by less adherence to infection prevention and control procedures. Social distancing, travel restriction measures, and reduced antibiotic drug use in the community during the pandemic might have prevented further ESBL-PE increases in community settings. Wastewater ESBL-PE surveillance may serve as a noninvasive, sensitive, rapid, and cost-effective instrument for early detection and monitoring the local epidemiology of ESBL-PE.

Appendix Methods and additional data for extended-spectrum β-lactamase–producing Enterobacterales in municipal wastewater collections, Switzerland, 2019–2023.
