# Supplementary material for: Extended-Spectrum β-Lactamase–Producing Enterobacterales in Municipal Wastewater Collections, Switzerland, 2019–2023
Source: Emerg Infect Dis. 2025 Mar;31(3):574–8. doi: 10.3201/eid3103.240099 (PMC11878307; doi:10.3201/eid3103.240099)
Supplement: Appendix — Methods and additional data for extended-spectrum β-lactamase–producing Enterobacterales in municipal wastewater collections, Switzerland, 2019–2023. [file 24-0099-Techapp-s1.pdf]

Article DOI: https://doi.org/10.3201/eid3103.240099

EID cannot ensure accessibility for supplementary materials supplied by authors. Readers who have difficulty accessing supplementary content should contact the authors for assistance.

Extended-Spectrum β-Lactamase–Producing Enterobacterales in Municipal Wastewater Collections, Switzerland, 2019–2023

Appendix

Supplemental Methods

In our 2023 publication, we described a wastewater surveillance network throughout the city of Basel, Switzerland (*1*), which allowed us to identify, and in some cases quantify, presumptive extended-spectrum β-lactamase–producing Enterobacterales (ESBL-PE) circulating in the city sewage system. Municipal wastewater samples were taken once a month from the sewage pipeline system covering 44% of the population of Basel at 21 different sampling points representing the 10 postal codes of Basel. Of those sampling points, 4 sites also collected wastewater from hospitals (4056/1, 4056/2, 4058/2, and 4051/3). Specifically, 4051/3 included 35%–40% of wastewater from the University Hospital Basel. The specific locations of the sampled sewers are provided elsewhere (*1*). Sampling sites were categorized on the basis of the wastewater sources received as urban (representing the community without wastewater from healthcare settings) and mixed (representing both community and healthcare settings) (Appendix Table 1). Samples were collected directly from the sewage system by the Civil Engineering Department of the Canton of Basel-Stadt following the specific recommendations of the World Health Organization (https://www.who.int/teams/environment-climate-change-and-health/water-sanitation-and-health/sanitation-safety). Because our study was exploratory and observational, no formal sample size calculation was performed.

Wastewater samples were collected in sterile 50-mL Falcon tubes (VWR, Dietikon) and transported within 6 hours in an insulated box to avoid warming. For the quantification, samples

were analyzed for the presence of presumptive ESBL-PE by plating without a previous enrichment step. For this, 100 µL of the samples was spread directly in a 1:10 dilution (Tryptic soy broth) on Brilliance ESBL agar (Oxoid; Thermo Fisher Scientific). Colony counting was performed after 24-hour incubation at 37°C, and categories were assigned according to their color on the basis of the manufacturer's manual. We distinguished 2 chromogenic groups for colonies: *E. coli* as blue/pink and the KESC group as green.

The details of all collected samples and the bacterial counts are shown in Appendix Table 2. We aimed to compare presumptive ESBL-PE abundance before (2019), during (2021), and after (2023) the COVID-19 pandemic.

Reference

1. Gómez-Sanz E, Bagutti C, Roth JA, Alt Hug M, García-Martín AB, Maurer Pekerman L, et al. Spatiotemporal dissemination of ESBL-producing Enterobacterales in municipal sewer systems: a prospective, longitudinal study in the city of Basel, Switzerland. Front Microbiol. 2023;14:1174336. PubMed https://doi.org/10.3389/fmicb.2023.1174336

Appendix Table 1. Overview of sample collection dates for extended-spectrum β-lactamase–producing Enterobacterales in municipal wastewater collections, Switzerland, 2019–2023*

Month	2018	2019	2021	2023
January		X		
February		X		
March		X		
April		X	X	X
May		X	X	X
June		X	X	X
July		X		
August	X			
September	X			
October	X			
November	X			
December	X			

*X notes when collections were made.

Appendix Table 2. Number of samples taken per sampling round stratified by sample source for extended-spectrum β-lactamase–producing Enterobacterales in municipal wastewater collections, Switzerland, 2019–2023

	Sample source		
Sampling period	Mixed	Urban	Total
2019*	4	17	21
May	4	17	21
April	4	17	21
June			
2021	4	17	21
May	3†	17	20
April	4	17	21
June			
2023	4	17	21
May	4	17	21
April			
June	4	17	21
Total	35	153	188

*Retrospective samples included from (*1*).
†One sample from postal code 4056 could not be taken.

Appendix Table 3. Mean CFU counts for extended-spectrum β-lactamase–producing Enterobacterales in municipal wastewater collections, Switzerland, 2019–2023*

Sample no.	Sampling sites, postal code/no.	Source	Mean *E. coli,* CFU/mL	Mean KESC, CFU/mL	Mean *E. coli* plus KESC, CFU/mL	Sampling month	Year of collection
001	4001/1	**Urban**	0	115	115	April	2019
002	4001/2	**Urban**	20	0	20	April	2019
003	4051/1	**Urban**	**750**	110	**860**	April	2019
004	4051/2	**Urban**	50	0	50	April	2019
005	4052/1	**Urban**	90	0	90	April	2019
006	4052/2	**Urban**	380	5	385	April	2019
007	4053/1	**Urban**	55	70	125	April	2019
008	4053/2	**Urban**	375	0	375	April	2019
009	4054/1	**Urban**	140	**510**	650	April	2019
010	4054/2	**Urban**	0	5	5	April	2019
011	4055/1	**Urban**	105	**9,700**	**9,805**	April	2019
012	4055/2	**Urban**	0	0	0	April	2019
013	4056/1	*Mixed*	455	120	575	April	2019
014	4056/2	*Mixed*	195	85	280	April	2019
015	4057/1	**Urban**	0	110	110	April	2019
016	4057/2	**Urban**	**2,595**	0	**2,595**	April	2019
017	4058/1	**Urban**	**1,905**	**665**	**2,570**	April	2019
018	4058/2	*Mixed*	120	15	135	April	2019
019	4059/1	**Urban**	100	160	260	April	2019
020	4059/2	**Urban**	280	**365**	645	April	2019
021	4051/3	*Mixed*	15	15	30	April	2019
022	4001/1	**Urban**	10	125	135	May	2019
023	4001/2	**Urban**	135	0	135	May	2019
024	4051/1	**Urban**	25	0	25	May	2019
025	4051/2	**Urban**	0	155	155	May	2019
026	4052/1	**Urban**	5	5	10	May	2019
027	4052/2	**Urban**	5	0	5	May	2019
028	4053/1	**Urban**	75	80	155	May	2019
029	4053/2	**Urban**	165	55	220	May	2019
030	4054/1	**Urban**	50	70	120	May	2019
031	4054/2	**Urban**	15	0	15	May	2019
032	4055/1	**Urban**	65	65	130	May	2019
033	4055/2	**Urban**	0	0	0	May	2019
034	4056/1	*Mixed*	205	15	220	May	2019
035	4056/2	*Mixed*	45	*840*	*885*	May	2019
036	4057/1	**Urban**	0	0	0	May	2019
037	4057/2	**Urban**	**1,655**	25	**1,680**	May	2019
038	4058/1	**Urban**	**1,385**	0	**1,385**	May	2019
039	4058/2	*Mixed*	60	0	60	May	2019
040	4059/1	**Urban**	185	145	330	May	2019
041	4059/2	**Urban**	10	70	80	May	2019
042	4051/3	*Mixed*	25	*235*	260	May	2019
043	4001/1	**Urban**	5	100	105	June	2019
044	4001/2	**Urban**	85	0	85	June	2019

Sample no.	Sampling sites, postal code/no.	Source	Mean *E. coli,* CFU/mL	Mean KESC, CFU/mL	Mean *E. coli* plus KESC, CFU/mL	Sampling month	Year of collection
045	4051/1	**Urban**	95	0	95	June	2019
046	4051/2	**Urban**	**15,480**	0	**15,480**	June	2019
047	4052/1	**Urban**	10	60	70	June	2019
048	4052/2	**Urban**	5	0	5	June	2019
049	4053/1	**Urban**	55	0	55	June	2019
050	4053/2	**Urban**	120	0	120	June	2019
051	4054/1	**Urban**	65	0	65	June	2019
052	4054/2	**Urban**	0	0	0	June	2019
053	4055/1	**Urban**	165	10	175	June	2019
054	4055/2	**Urban**	0	0	0	June	2019
055	4056/1	*Mixed*	225	30	255	June	2019
056	4056/2	*Mixed*	5	0	5	June	2019
057	4057/1	**Urban**	5	10	15	June	2019
058	4057/2	**Urban**	230	55	285	June	2019
059	4058/1	**Urban**	175	10	185	June	2019
060	4058/2	*Mixed*	40	45	85	June	2019
061	4059/1	**Urban**	5	0	5	June	2019
062	4059/2	**Urban**	10	0	10	June	2019
063	4051/3	*Mixed*	425	5	430	June	2019
064	4001/1	Urban	**1,025**	110	1,135	April	2021
065	4001/2	Urban	**1,215**	1,080	2,295	April	2021
066	4051/1	Urban	40	960	1,000	April	2021
067	4051/2	Urban	0	0	0	April	2021
068	4052/1	Urban	155	285	440	April	2021
069	4052/2	Urban	235	70	305	April	2021
070	4053/1	Urban	190	850	1040	April	2021
071	4053/2	Urban	310	225	535	April	2021
072	4054/1	Urban	115	125	240	April	2021
073	4054/2	Urban	475	0	475	April	2021
074	4055/1	Urban	190	450	640	April	2021
075	4055/2	Urban	0	0	0	April	2021
076	4056/1	Mixed	1,780	*1,800*	3,580	April	2021
077	4057/1	Urban	50	55	105	April	2021
078	4057/2	Urban	395	605	1,000	April	2021
079	4058/1	Urban	50	**3,420**	**3,470**	April	2021
080	4058/2	Mixed	895	810	1705	April	2021
081	4059/1	Urban	240	210	450	April	2021
082	4059/2	Urban	170	40	210	April	2021
083	4051/3	Mixed	40	190	230	April	2021
084	4001/1	Urban	220	225	445	May	2021
085	4001/2	Urban	195	305	500	May	2021
086	4051/1	Urban	30	20	50	May	2021
087	4051/2	Urban	145	50	195	May	2021
088	4052/1	Urban	0	50	50	May	2021
089	4052/2	Urban	20	20	40	May	2021
090	4053/1	Urban	**1,330**	115	1,445	May	2021
091	4053/2	Urban	**15,400**	**2,900**	**18,300**	May	2021
092	4054/1	Urban	245	60	305	May	2021
093	4054/2	Urban	10	10	20	May	2021
094	4055/1	Urban	10	130	140	May	2021
095	4055/2	Urban	10	250	260	May	2021
096	4056/1	Mixed	625	425	1,050	May	2021
097	4056/2	Mixed	420	705	1,125	May	2021
098	4057/1	Urban	0	10	10	May	2021
099	4057/2	Urban	235	225	460	May	2021
100	4058/1	Urban	0	**8,400**	**8,400**	May	2021
101	4058/2	Mixed	245	645	890	May	2021
102	4059/1	Urban	**1,460**	155	1,615	May	2021
103	4059/2	Urban	420	40	460	May	2021
104	4051/3	Mixed	330	140	470	May	2021
105	4001/1	Urban	150	925	1,075	June	2021
106	4001/2	Urban	10	165	175	June	2021
107	4051/1	Urban	60	10	70	June	2021
108	4051/2	Urban	0	40	40	June	2021
109	4052/1	Urban	200	**11,400**	**11,600**	June	2021
110	4052/2	Urban	170	20	190	June	2021
111	4053/1	Urban	535	80	615	June	2021
112	4053/2	Urban	**845**	740	1,585	June	2021

Sample no.	Sampling sites, postal code/no.	Source	Mean *E. coli,* CFU/mL	Mean KESC, CFU/mL	Mean *E. coli* plus KESC, CFU/mL	Sampling month	Year of collection
113	4054/1	Urban	140	160	300	June	2021
114	4054/2	Urban	60	130	190	June	2021
115	4055/1	Urban	230	80	310	June	2021
116	4055/2	Urban	80	800	880	June	2021
117	4056/1	Mixed	*4,200*	*3,900*	*8,100*	June	2021
118	4056/2	Mixed	*3,300*	615	3,915	June	2021
119	4057/1	Urban	**18,100**	0	**18,100**	June	2021
120	4057/2	Urban	**6,170**	**1,825**	**7,995**	June	2021
121	4058/1	Urban	190	**7,405**	**7,595**	June	2021
122	4058/2	Mixed	1,075	930	2,005	June	2021
123	4059/1	Urban	40	20	60	June	2021
124	4059/2	Urban	60	105	165	June	2021
125	4051/3	Mixed	770	505	1,275	June	2021
126	4001/1	Urban	160	90	250	April	2023
127	4001/2	Urban	20	15	35	April	2023
128	4051/1	Urban	10	15	25	April	2023
129	4051/2	Urban	55	15	70	April	2023
130	4052/1	Urban	630	5	635	April	2023
131	4052/2	Urban	65	**2,745**	2,810	April	2023
132	4053/1	Urban	275	200	475	April	2023
133	4053/2	Urban	**1,525**	180	1,705	April	2023
134	4054/1	Urban	860	40	900	April	2023
135	4054/2	Urban	475	5	480	April	2023
136	4055/1	Urban	**10,375**	35	**10,410**	April	2023
137	4055/2	Urban	55	10	65	April	2023
138	4056/1	Mixed	330	420	750	April	2023
139	4056/2	Mixed	845	610	1,455	April	2023
140	4057/1	Urban	65	15	80	April	2023
141	4057/2	Urban	**1,385**	420	1,805	April	2023
142	4058/1	Urban	1,160	**2,100**	3,260	April	2023
143	4058/2	Mixed	795	245	1,040	April	2023
144	4059/1	Urban	25	55	80	April	2023
145	4059/2	Urban	40	**3,830**	**3,870**	April	2023
146	4051/3	Mixed	140	15	155	April	2023
147	4001/1	Urban	45	5	50	May	2023
148	4001/2	Urban	410	**1,475**	1,885	May	2023
149	4051/1	Urban	0	5	5	May	2023
150	4051/2	Urban	60	0	60	May	2023
151	4052/1	Urban	**1,515**	440	1,955	May	2023
152	4052/2	Urban	470	260	730	May	2023
153	4053/1	Urban	495	240	735	May	2023
154	4053/2	Urban	150	280	430	May	2023
155	4054/1	Urban	225	10	235	May	2023
156	4054/2	Urban	**125,300**	0	**125,300**	May	2023
157	4055/1	Urban	195	190	385	May	2023
158	4055/2	Urban	90	20	110	May	2023
159	4056/1	Mixed	805	630	1435	May	2023
160	4056/2	Mixed	500	*4,595*	*5,095*	May	2023
161	4057/1	Urban	35	5	40	May	2023
162	4057/2	Urban	205	55	260	May	2023
163	4058/1	Urban	100	15	115	May	2023
164	4058/2	Mixed	310	780	1,090	May	2023
165	4059/1	Urban	60	0	60	May	2023
166	4059/2	Urban	60	0	60	May	2023
167	4051/3	Mixed	85	*5,550*	*5,635*	May	2023
168	4001/1	Urban	670	110	780	June	2023
169	4001/2	Urban	10	15	25	June	2023
170	4051/1	Urban	140	0	140	June	2023
171	4051/2	Urban	5	**2,150**	2,155	June	2023
172	4052/1	Urban	10	10	20	June	2023
173	4052/2	Urban	110	325	435	June	2023
174	4053/1	Urban	745	245	990	June	2023
175	4053/2	Urban	250	50	300	June	2023
176	4054/1	Urban	100	115	215	June	2023
177	4054/2	Urban	0	145	145	June	2023
178	4055/1	Urban	**2,900**	125	3,025	June	2023
179	4055/2	Urban	80	0	80	June	2023
180	4056/1	Mixed	750	275	1,025	June	2023

Sample no.	Sampling sites, postal code/no.	Source	Mean *E. coli*, CFU/mL	Mean KESC, CFU/mL	Mean *E. coli* plus KESC, CFU/mL	Sampling month	Year of collection
181	4056/2	Mixed	*3,490*	*3,705*	*7,195*	June	2023
182	4057/1	Urban	5	40	45	June	2023
183	4057/2	Urban	**1,765**	280	2,045	June	2023
184	4058/1	Urban	1,185	**1,815**	3,000	June	2023
185	4058/2	Mixed	530	640	1,170	June	2023
186	4059/1	Urban	25	5	30	June	2023
187	4059/2	Urban	25	10	35	June	2023
188	4051/3	Mixed	255	275	530	June	2023

*Mean CFU counts for *Escherichia coli* alone, *Klebsiella*, *Enterobacter*, *Serratia*, and *Citrobacter* (KESC) group, and all together (*E. coli* plus KESC) stratified by collection point (postal codes) and sampling month before, during, and after the COVID pandemic. Outliers according to period are indicated in the corresponding font colors (green, red, or blue); outliers according to source are indicated in the corresponding font style (italic or bold). Outlier were calculated on the basis of the interquartile range method.

Appendix Table 4. Quantification across 3 years of extended-spectrum β-lactamase–producing Enterobacterales in municipal wastewater collections, Switzerland, 2019–2023, stratified by month*

Characteristics	ESBL *E. coli*	ESBL KESC	ESBL *E. coli* + KESC
April 2019			
Median CFU/mL (IQR)	105 (20–375)	70 (0−120)	260 (90–645)
Range, CFU/mL	0−2,595	0−9,700	0−9,805
April 2021			
Median CFU/mL (IQR)	190 (50−435)	218 (63−830)	505 (235−1,088)
Range, CFU/mL	0−1,780	0−3,420	0−3,580
April 2023			
Median CFU/mL (IQR)	275 (55−845)	55 (15−420)	635 (80−1,705)
Range, CFU/mL	10−10,375	5−3,830	25−10,410
April p value	0.332	0.069	0.228
May 2019			
Median CFU/mL (IQR)	45 (10–135)	25 (0−80)	135 (25–220)
Range, CFU/mL	0−1,655	0−840	0−1,680
May 2021			
Median CFU/mL (IQR)	220 (10−420)	140 (50–305)	460 (140−1,050)
Range, CFU/mL	0−14,500	10−8,400	10−18,300
May 2023			
Median CFU/mL (IQR)	195 (60–470)	55 (5−440)	385 (60−1,435)
Range, CFU/mL	0−125,300	0–5,550	5−125,300
May p value	**0.027**	**0.023**	**0.042**
June 2019			
Median CFU/mL (IQR)	55 (5–165)	0 (0–10)	85 (10–175)
Range, CFU/mL	0−15,480	0–100	0−15,480
June 2021			
Median CFU/mL (IQR)	190 (60–845)	165 (80–925)	880 (190−3,915)
Range, CFU/mL	0−18,100	0−11,400	40−18,100
June 2023			
Median CFU/mL (IQR)	140 (25–745)	125 (15–280)	435 (80−1,170)
Range, CFU/mL	0−3,490	0–3,705	20−7,195
June p value	**0.025**	**<0.001**	**<0.001**

*Sample size, n = 21 except for ESBL-KESC and ESBL *E. coli* + KESC in April 2021, where the sample size was n = 20. Bold indicates statistically significant values. p values represent 2019 versus 2021 versus 2023 by Kruskal-Wallis test. ESBL, extended-spectrum β-lactamase; IQR, interquartile range; KESC, *Klebsiella, Enterobacter, Serratia,* and *Citrobacter* group.

Appendix Table 5. Quantification across 3 years of presumptive extended-spectrum β-lactamase–producing Enterobacterales in municipal wastewater collections, Switzerland, 2019–2023, stratified by source and by month*

	ESBL *E. coli*		ESBL KESC		ESBL *E. coli* + KESC	
Characteristics	Mixed	Urban	Mixed	Urban	Mixed	Urban
April 2019						
Median CFU/mL (IQR)	158 (68−325)	100 (20−375)	50 (15−103)	70 (0−160)	208 (83−428)	160 (90−650)
Range, CFU/mL	15−455	0−2,595	15−120	0−9,700	30−575	0−9,805
p value	0.653		0.786		0.788	
April 2021						
Median CFU/mL (IQR)	895 (40−1,780)	190 (50−310)	810 (190−1,800)	210 (55–605)	1705 (230−3,580)	475 (240−1,000)
Range, CFU/mL	40−1,780	0−1,215	190−,1800	0−3,420	230−3,580	0−3,470
p value	0.340		0.223		0.266	
April 2023						
Median CFU/mL (IQR)	563 (235−820)	160 (55−860)	333 (130–515)	40 (15−200)	895 (453−1,248)	480 (80−1,805)
Range, CFU/mL	140−845	10−10,375	15−610	5−3,830	155−1,455	25−10,410
p value	0.473		0.230		0.720	
May 2019						
Median CFU/mL (IQR)	53 (35−133)	100 (20−375)	125 (8−538)	25 (0−70)	240 (140−573)	130 (15−155)
Range, CFU/mL	25−205	0−2,595	0–840	0−155	60−885	0−1,680
p value	0.475		0.289		0.194	
May 2021						
Median CFU/mL (IQR)	375 (288−523)	145 (10−245)	535 (283−675)	115 (40–225)	970 (680−1,088)	305 (50−500)
Range, CFU/mL	245−625	0−15,400	140−705	10−8,400	470−1,125	10−18,300
p value	0.088		0.069		0.128	
May 2023						
Median CFU/mL (IQR)	405 (198−653)	150 (60−410)	2,688 (705–5,073)	15 (5−240)	3,265 (1,263−5,365)	235 (60−730)
Range, CFU/mL	85−805	0−125,300	630−5,550	0−1,475	1,090−5,635	5−125,300
p value	0.209		**0.004**		**0.020**	
June 2019						
Median CFU/mL (IQR)	133 (23−325)	55 (5−120)	18 (3–38)	0 (0−10)	170 (45−343)	70 (10−120)
Range, CFU/mL	5−425	0−15,480	0−45	0−100	5−430	0−15,480
p value	0.417		0.297		0.394	
June 2021						
Median CFU/mL (IQR)	2,188 (923−3,750)	150 (60−230)	773 (560−2,415)	130 (40−800)	2,960 (1,640−6,008)	310 (175−1,585)
Range, CFU/mL	770−4,200	0−18,100	505−3,900	0−11,400	1,275−8,100	40−18,100
p value	**0.025**		0.128		0.089	
June 2023						
Median CFU/mL (IQR)	640 (393−2,120)	100 (10−670)	458 (275−2,173)	110 (10−245)	1,098 (778−4,183)	215 (45−990)
Range, CFU/mL	255−3,490	0−2,900	275−3,705	0−2,150	530−7,195	20−3,025
p value	0.060		0.031		0.073	

*Sample size: n = 4 for Mixed and n = 17 for Urban, except for April 2021, where the sample size was n = 3 for Mixed. Mixed versus urban by Mann-Whitney U test. Bold indicates statistically significant values. ESBL, extended-spectrum β-lactamase; IQR, interquartile range; KESC, *Klebsiella, Enterobacter, Serratia,* and *Citrobacter* group.

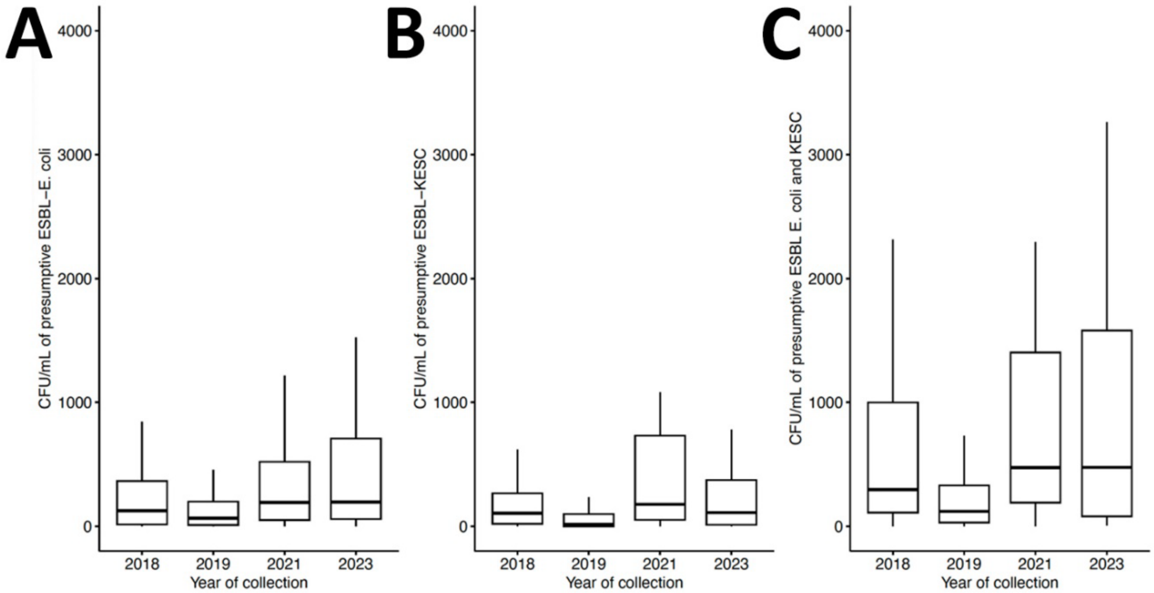

Appendix Figure 1. Temporal quantification of presumptive extended-spectrum β-lactamase (ESBL)-producing *E. coli* and *Klebsiella*, *Enterobacter*, *Serratia*, and *Citrobacter* (KESC) group colonies. (A–C) Data points from all sampling months across the 21 sampling points distributed across Basel (representing 44% of Basel population) are collapsed and represented per year. (A) Temporal distribution of presumptive ESBL-producing *E. coli*, (B) presumptive ESBL-producing KESC, and (C) presumptive ESBL-producing *E. coli* plus KESC across the sampling years. In all graphs (A–C), outliers were removed for readability. Boxes, bold lines, and whiskers indicate the interquartile ranges, medians, and 1.5 times the interquartile range, respectively.

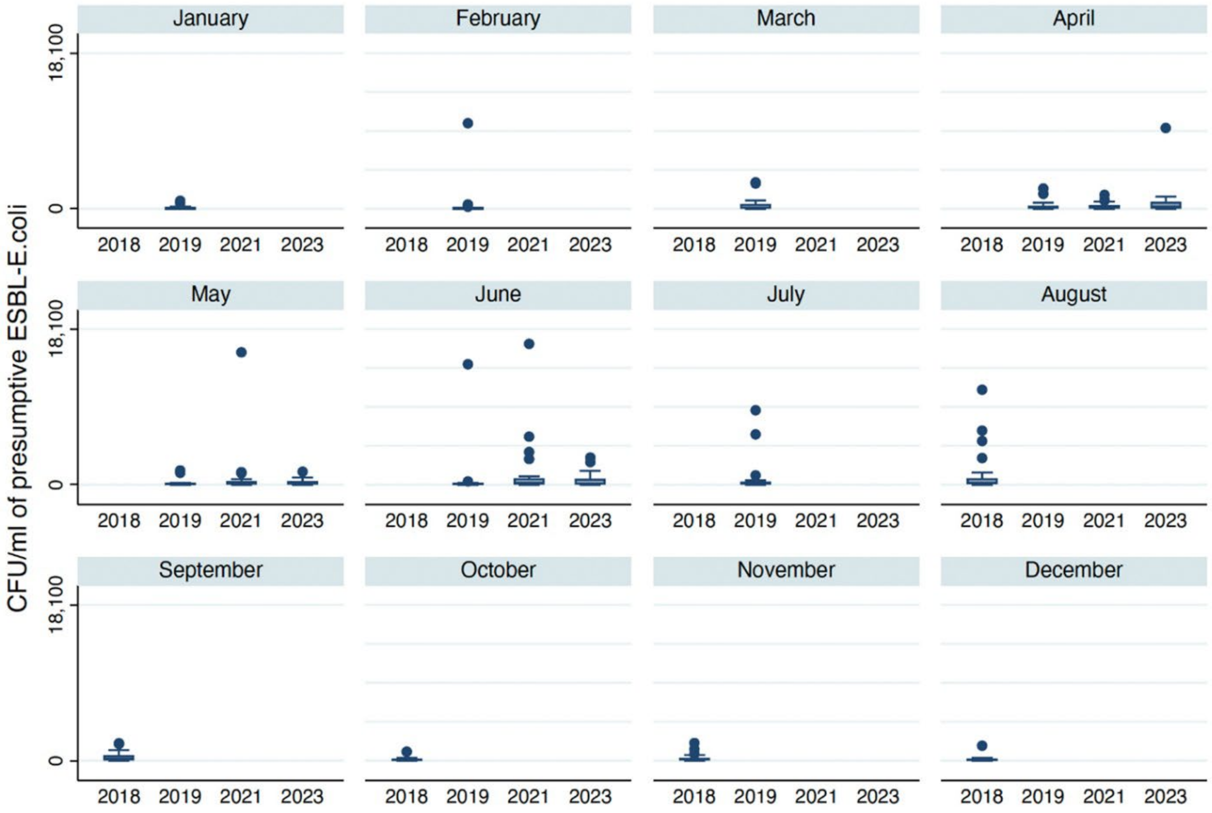

Appendix Figure 2. Counts (CFU/mL) of presumptive ESBL *E. coli* per sampling year stratified by month during the entire study period (excluding 1 value with >20,000 CFU collected in May 2023 for better visualization).

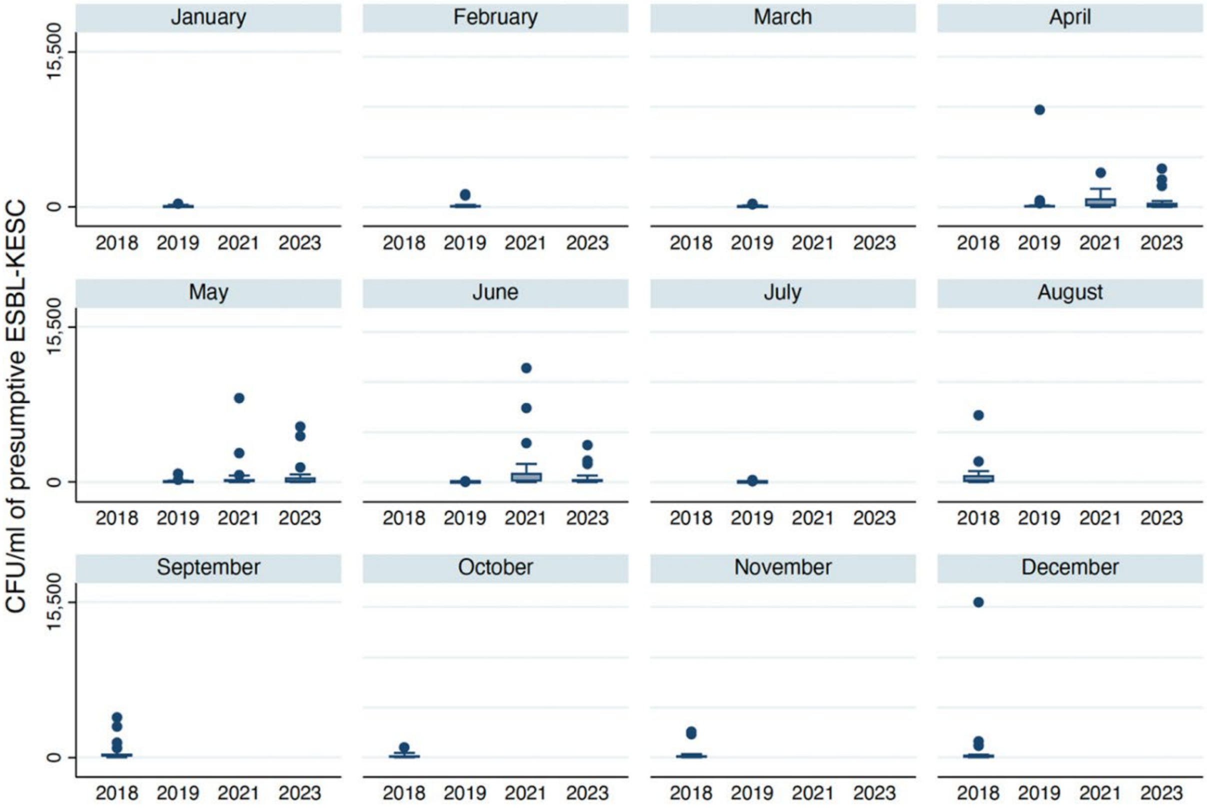

Appendix Figure 3. Counts (CFU/mL) of presumptive ESBL *Klebsiella, Enterobacter, Serratia,* and *Citrobacter* (KESC) group per sampling year stratified by month during the entire study period.

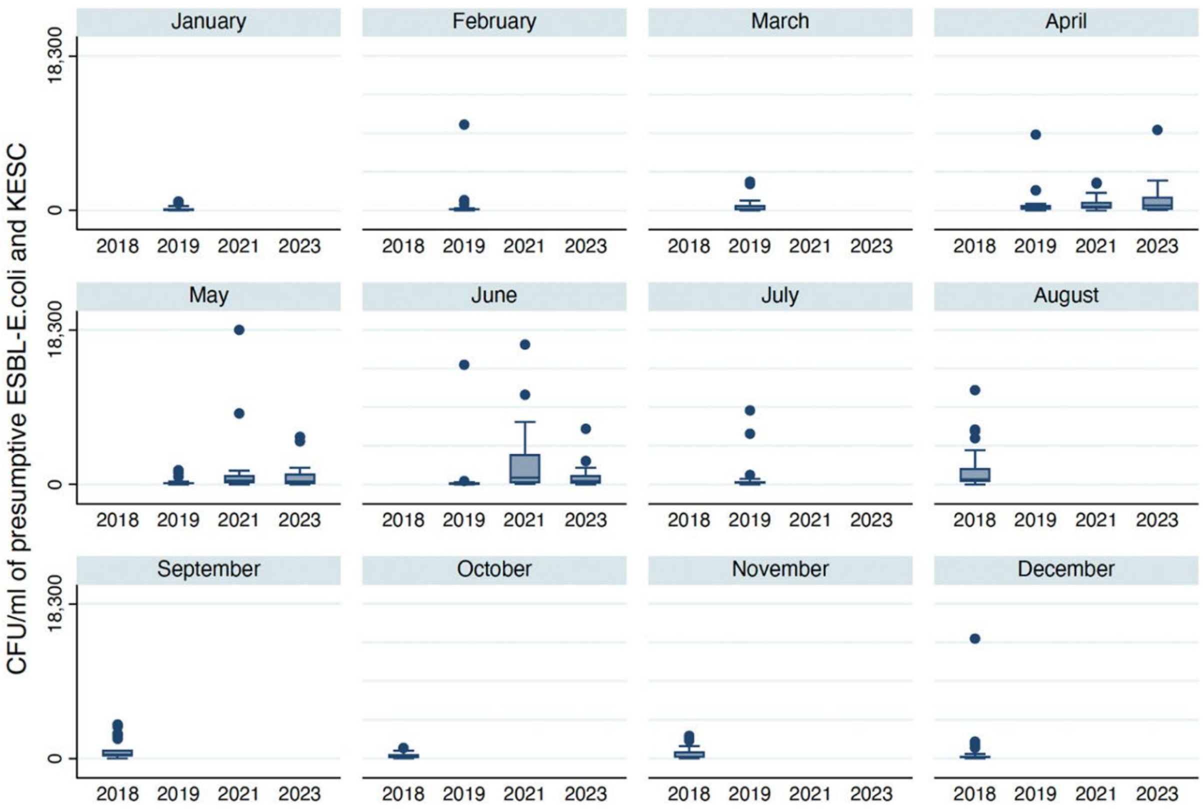

Appendix Figure 4. Counts (CFU/mL) of presumptive ESBL *Klebsiella, Enterobacter, Serratia*, and *Citrobacter* (KESC) group per sampling year stratified by month during the entire study period (excluding 1 value with >20,000 CFU collected in May 2023 for better visualization).
